# Apical Pressure Generated Using Conventional Syringe Irrigation in Immature Teeth—An In Vitro Study

**DOI:** 10.3390/ma14102580

**Published:** 2021-05-15

**Authors:** Marco Jäggi, Eva Magni, Florin Eggmann, Ashraf ElAyouti, Thomas Connert, Roland Weiger

**Affiliations:** 1 Department of Reconstructive Dentistry, University Center for Dental Medicine Basel UZB, University of Basel, 4058 Basel, Switzerland; marco.jaeggi@unibas.ch; 2 Department of Periodontology, Endodontology and Cariology, University Center for Dental Medicine Basel UZB, University of Basel, 4058 Basel, Switzerland; eva.magni@unibas.ch (E.M.); thomas.connert@unibas.ch (T.C.); roland.weiger@unibas.ch (R.W.); 3 Division of Endodontology, Department of Conservative Dentistry, School of Dental Medicine, Eberhard Karls University of Tübingen, 72076 Tübingen, Germany; ashraf.elayouti@med.uni-tuebingen.de

**Keywords:** endodontics, NaOCl accident, open apex, root canal treatment, sodium hypochlorite, dental trauma

## Abstract

This in vitro study aimed to evaluate apical pressure during irrigant delivery with syringe irrigation in immature teeth with an open apical foramen. Conventional syringe irrigation was performed in a 3D-printed immature incisor. A 5 mL syringe combined with 25 G and 30 G cannulas was used. Open-ended and side-vented needle tip designs were assessed. Cannulas were placed at tooth length (TL), TL −1 mm, TL −2 mm, and TL −4 mm. The syringe plunger was moved with a force of 10 N, 20 N, 40 N, and 80 N to simulate clinical conditions. A pressure sensor measured periapical pressures during irrigation. Each experiment was repeated 10 times. Data were analyzed descriptively (maximum, mean, standard deviation, 95% CI) with the critical threshold indicative of extrusion set at 7.64 mbar. 30 G cannulas with both needle tip designs never exceeded the threshold at any TL with a plunger force of 10–40 N. At 80 N, 30 G open-ended cannulas exceeded the threshold in 10%, 30 G side-vented in 20–60% of the measurements. At any TL, 25 G open-ended cannulas and 25 G side-vented cannulas never crossed the threshold with forces of 10–20 N and 10 N, respectively. Consequently, 30 G cannulas with both designs can be recommended for irrigant delivery in immature teeth. 25 G cannulas ought to be used with caution.

## 1. Introduction

Irrigation of the root canal system with chemical agents is an essential part of root canal treatment. Irrigation with sodium hypochlorite (NaOCl) supplements the removal of pulp tissue and microorganisms. Other irrigants, such as ethylenediaminetetraacetic acid, can remove the smear layer, which results from the mechanical preparation of root canal walls [1]. Today various techniques like ultrasonic irrigant activation are available to enhance irrigation [2]. However, to deliver irrigants during root canal treatment, syringe irrigation is the most commonly used method [3,4].

NaOCl, the most widely used irrigant, can dissolve remnants of pulpal tissue and organic components of dentin [3,4,5,6]. However, NaOCl exerts cytotoxic effects in contact with healthy tissues. NaOCl accidents caused by apical irrigant extrusion are rare in clinical practice, but they can entail severe consequences for affected patients [7].

Immediately after a NaOCl accident, some patients feel intense pain and display swelling in the face [7]. In addition, owing to vasodilatation, hemorrhage into the interstitial tissue (ecchymosis) and edema may occur. Other symptoms described in case reports include, without limitation, ulcers of the skin, swelling expanding to the periorbital region or towards the sternum, anemic skin areas, a chlorine-like odor in the nose, irritation of the throat, and breathing difficulties [8,9,10,11]. In most cases, these signs and symptoms resolve within a month, but patients may experience persistent paresthesia or permanent loss of sensory function when the NaOCl accident inflicted nerve damage [9,12,13].

Typical distribution patterns of ecchymosis involve the angle of the mouth, the periorbital region, or the neck down to the sternum. Such ecchymosis may occur when NaOCl enters the venous system [7]. It is presumed that apically extruded NaOCl must exceed the central venous pressure (5.73 mmHg = mean-SD of 0.15 mmHg) to enter into the venous system [7,14,15]. 5.58 mmHg (lower level of the central venous pressure), corresponding to 7.64 mbar, was therefore chosen as the critical value to investigate apical irrigant extrusion in previous studies [16,17,18]. However, the apical tissue pressure countering an extrusion is another important factor that varies among individuals and depends on the periapical status [19].

The factors that play a role in apical irrigant extrusion are legion. For instance, wedging of the irrigation needle can promote NaOCl extrusion [20]. Moreover, apical root resorption and a large apical foramen are widely thought to increase the risk of irrigant extrusion [21]. The size of the apical foramen can be assessed radiographically with two-dimensional radiographs and, more accurately, with cone-beam computed tomography [22]. To reduce the likelihood of extrusion incidents, side-vented cannulas have been recommended to deliver irrigants to the apical portion of a root canal [11]. This recommendation is based on the observation that apical pressures decrease when side-vented cannulas are used instead of open-ended cannulas [23]. Furthermore, operator-related factors, such as the force applied to the plunger of a syringe and the insertion depth of the irrigation needle, have a determining influence on the apical pressure and hence the risk of irrigant extrusion [9,23].

Case reports aside, data on irrigant extrusion in teeth with a wide-open apex are currently scanty. Therefore, this in vitro study aimed to evaluate the apical pressure of NaOCl in an immature tooth with an open apical foramen using conventional syringe irrigation at different needle insertion depths.

## 2. Materials and Methods

### 2.1. Preliminary Experiment

To assess the forces exerted on the plunger of an irrigation syringe, sixteen dentists, nine females and seven males, were tasked to perform syringe irrigation. A 5 mL syringe (Omnifix Solo, B. Braun Medical AG, Sempach, Switzerland) was used. The participants were instructed to undertake the procedure as they do when providing endodontic patient care. The participating dentists performed root canal irrigation on a 3D-printed tooth with the irrigation cannula placed at a working length of 16 mm as follows: twice, for at least 10 s, with a 30 G side-vented cannula, and twice, again for at least 10 s, with a 25 G side-vented cannula. Water was used as irrigant.

For these experiments, 64 in total, the plunger of the 5 mL syringe was fitted with a force sensor (KM25, Transmetra GmbH, Flurlingen, Switzerland), which had a recording range of 0–100 N. The force sensor was connected to a measurement amplifier (GM40, Lorenz Messtechnik GmbH, Alfdorf, Germany) by a plug-in data cable. The amplifier relayed the signal of the force sensor to an analog–digital converter (12 bit, RedLab 1208FS USB Mini-Messlabor, Meilhaus Electronic GmbH, Alling, Germany). The converted digital data were then transferred to a personal computer and processed with laboratory software (LabVIEW, International Instruments, Austin, TX, USA).

Maximum forces between 3 and 51 N were recorded during these experiments. Based on these data, standardized plunger forces were selected for the comprehensive in vitro assessment of apical pressures during syringe irrigation.

### 2.2. Experimental Setup

**Figure 1:**
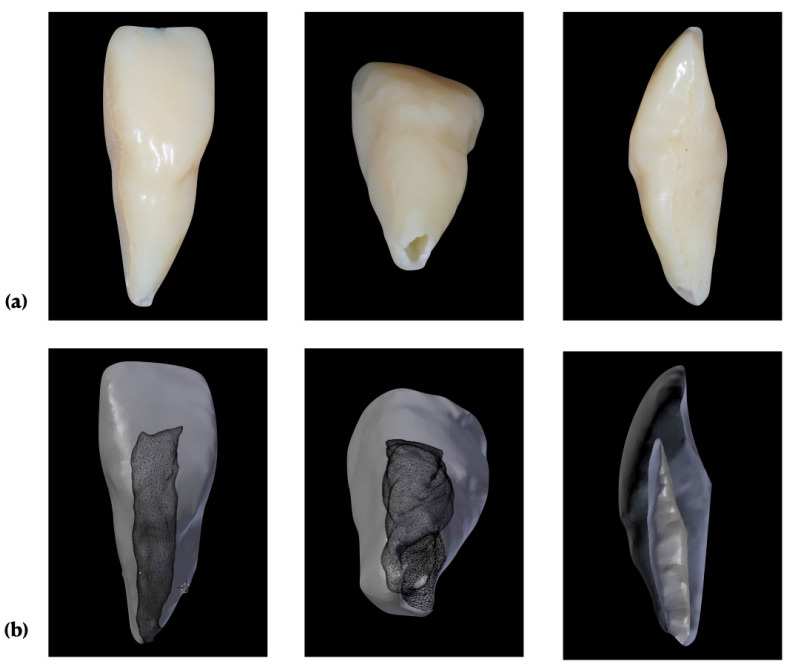
(**a**) 3D-printed central maxillary incisor, view from buccal, apical, and longitudinal section; (**b**) 3D rendering based on a micro-computed tomography imaging.

A 3D-printed central maxillary incisor was used for the experiments. The printed tooth was based on micro-computed tomography imaging data of an immature tooth #21. Its apical cross-section had a minimum diameter of 1.57 mm, a maximal diameter of 2.11 mm, a circumference of 5.84 mm, and an area of 2.49 mm^2^. The total distance from the incisal edge to the apex, henceforth referred to as tooth length (TL), was 17 mm. Figure 1 shows a picture of the immature tooth #21 and a 3D rendered (ITK-SNAPE, University of Pennsylvania, Philadelphia, PA, USA) image of the micro-computed tomography data.

**Figure 2:**
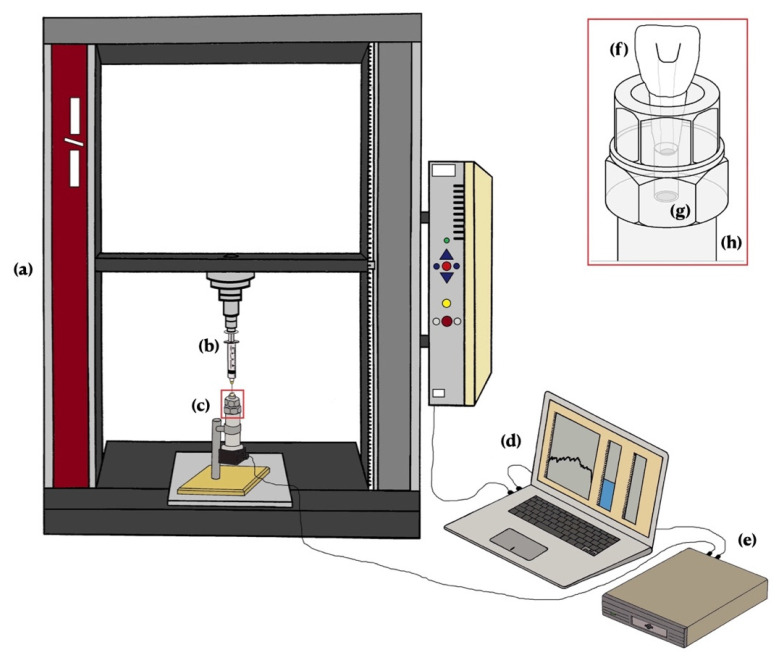
Experimental setup: (**a**) universal testing machine generating the force on the Scheme 30 G/25 G cannula. (**b**) Syringe filled with water and fitted with 30 G/25 G cannula. (**c**) 3D-printed central incisor on the pressure sensor. (**d**) Personal computer with laboratory software. (**e**) Analog–digital converter. (**f**) 3D-printed tooth fixed in a threaded cylinder with silicone sealant. (**g**) Pressure membrane (**h**) integrated into the pressure sensor.

The printed tooth was fixed in a threaded cylinder with silicone sealant (JBL AquaSil Schwarz, JBL GmbH & Co. KG, Neuhofen, Germany). The cylinder was placed on a pressure sensor (IMP320, ICS Schneider Messtechnik GmbH, Hohen Neuendorf, Germany) with a measurement range of 0–160 mbar. The pressure sensor had an integrated amplifier, generating analog output signals of 4–20 mA with a precision of 0.1%. The response time of the pressure sensor was ≤0.5 ms. The pressure sensor had a sampling rate of 10 kHz, which allowed dynamic measurements. The enclosed chamber between the pressure sensor and the apex of the tooth was filled with water. During irrigation, the generated pressures were directly transferred to the sensor without alteration owing to water’s lack of compressibility. The amplified signal from the pressure sensor was converted by an analog–digital converter and transferred to a personal computer. Data were processed with laboratory software (LabVIEW) with a customized protocol to read and save obtained data. Figure 2 shows the experimental setup.

### 2.3. Irrigation Methods

The irrigation experiments were carried out with a 5 mL single-use syringe (Omnifix Solo). The syringe was filled with water and mounted above the sensor using a fixation clamp. 25 G and 30 G cannulas (Transcodent GmbH & Co. KG, Kiel, Germany), both with open-ended and side-vented needle tip designs, were used. The 25 G open-ended cannula had an inner diameter of 0.25 mm with an open area of 0.05 mm^2^. The 30 G open-ended cannula had an inner diameter of 0.15 mm with an open area of 0.02 mm^2^. The 25 G side-vented cannula had an inner diameter of 0.25 mm and a lateral opening of 0.25 mm^2^ (1.0 mm × 0.25 mm). The 30 G side-vented cannula had an inner diameter of 0.15 mm and a lateral opening of 0.15 mm^2^ (1.0 mm × 0.15 mm). All measurements of the diameter and area of the irrigation cannulas were performed using light microscopy (Wild M7A, Leica, Heerbrugg, Switzerland). Root canal irrigation was performed at the following cannula insertion depths: TL, TL −1 mm, TL −2 mm, and TL −4 mm. To ensure consistent force application, a universal testing machine (Z020, ZwickRoell GmbH & Co. KG, Ulm, Germany) was employed to move the syringe plunger. Based on the preliminary experiment, the force load was set to 10 N, 20 N, 40 N, and 80 N. Testing was repeated 10 times for all combinations of plunger force, cannula size, needle tip design, and insertion depth. Depending on the force and the diameter of the needle, testing took between 4 and 30 s. The volume of irrigant delivered during irrigation was recorded in all runs of the irrigation experiments.

### 2.4. Critical Values and Statistics

A descriptive statistical analysis was performed. The maximum pressure values of each measurement and the 95% confidence intervals were subjected to the analysis. To gauge the risk of apical NaOCl extrusion, the mean central venous pressure of 7.64 mbar was defined as a critical threshold. Pressure values exceeding this critical threshold were considered indicative of potential apical NaOCl extrusion.

## 3. Results

Table 1 lists the results of all measurements. With a plunger force ≤40 N, 30 G cannulas with both needle tip designs never exceeded the critical threshold at any insertion depth. With a plunger force of 80 N, 30 G open-ended cannulas and 30 G side-vented cannulas crossed the critical threshold in 10% and 20–60% of the measurements, respectively. Irrespective of the cannula insertion depth, no pressures indicative of irrigant extrusion were observed with 25 G open-ended cannulas and 25 G side-vented cannulas at plunger forces of 10–20 N and 10 N, respectively.

Irrespective of the insertion depth, 25 G open-end cannulas and 25 G side-vented cannulas crossed the critical threshold in 100% of the measurements at 80 N and 40–80 N, respectively.

A tendency was observed for cannulas with a side-vented needle tip design to produce higher apical pressures than open-ended cannulas. Table 2 shows clinical recommendations to perform irrigation in teeth with an open apex, provided under the caveat that the clinical conditions are similar to the reported experimental setup.

## 4. Discussion

This in vitro study comprehensively assessed apical irrigant pressures in an immature maxillary incisor with an open apex during conventional syringe irrigation. The main results of the investigation showed that 30 G open-ended and side-vented cannulas never exceeded the critical threshold of 7.64 mbar when plunger forces between 10 and 40 N were applied. Consequently, even when the irrigation needle is placed near working length, irrigant delivery with 30 G cannulas and a small to moderate plunger force involves little risk of apical irrigant extrusion. By contrast, irrigant delivery with 25 G cannulas, irrespective of their needle tip design, increases the risk of apical irrigant extrusion. Using plunger forces between 40 and 80 N, 25 G open-ended needles produced apical pressures indicative of apical irrigant extrusion. Data of the present study suggest that the plunger force required to exceed the critical threshold is even smaller with a side-vented needle tip design. 25 G side-vented cannulas exceeded the critical threshold when plunger forces ranging from 20–80 N were used. Thus, 25 G cannulas must be used with due caution regardless of their needle tip design.

This study is of clinical importance because NaOCl accidents, ranging from minor to severe, must be avoided, and conventional syringe irrigation is the most commonly used irrigant delivery method among general dental practitioners and endodontists alike [3,4]. The experimental setup allowed us to measure apical pressures that occur during root canal irrigation under simulated conditions. Irrigating needles with an open-ended and a side-vented design in sizes 30 G and 25 G were used because these cannula sizes are frequently chosen in clinical practice [23].

The setup and findings of the present study need to be interpreted in the context of the available body of evidence. A previous study reported that, at an equal distance from the apex, side-vented cannulas produce lower apical pressures compared with open-ended ones [23]. Contrary to this, the present study found only minor differences between these needle tip designs, and it was, in fact, side-vented cannulas that tended to produce higher apical pressures. Both cannula designs tested had the same inner diameter. Side-vented cannulas tended to have a higher flow rate compared with open-ended ones at the same plunger force. This may be explained by the Hagen–Poiseuille equation with a slight difference in the length of the cannulas. In addition, the fluid dynamics within the larger lumen of the main root canal of immature teeth may differ from root canals with a smaller diameter.

The strengths and inherent limitations of the present laboratory study, which assessed the apical pressure during irrigation, require careful consideration. The apical tissue pressure, likely varying between individuals, remains unknown. It depends, moreover, on the clinical condition [19]. Data from animal experiments suggest that the tissue fluid pressure at the apical foramen is unstable. Depending on the clinical situation, the apical fluid pressure can reach −15 mbar up to +45 mbar after pulp extirpation and −16 mbar up to 57 mbar in the presence of generalized edema [24]. It is currently unknown what apical pressure is needed for irrigant extrusion beyond the apex to occur. In the present study, the central venous pressure of 7.64 mbar was defined as a critical threshold because it was the lowest reference value relevant for apical irrigant extrusion reported in the literature [15,16]. It is, however, important to consider that previous studies used different pressure thresholds to investigate apical irrigant extrusion. Previously used pressure thresholds included the capillary pressure (25 mmHg), the interstitial pressure (20–30 mmHg), and the intraosseous blood pressure (30 mmHg) [16,17,18,19,25]. Using one of these thresholds instead of 7.64 mbar, the interpretation of data obtained in the present study is different. As the present study recorded apical pressures in mbar, it is possible to undertake the analysis with a different critical threshold if and when more evidence is available.

The flow rate of conventional syringe irrigation is controlled by the practitioner, who, as a consequence, plays an important role in the safe delivery of irrigating solutions [26]. The preliminary experiment assessed the force applied to the plunger of an irrigation syringe using a convenience sample of 16 dentists. Forces between 3 and 51 N were recorded, and the subsequent experiments revealed the force applied to the plunger of a syringe to have a determining influence on irrigant extrusion. Therefore, dental practitioners should be wary of delivering irrigants with excessive force: syringe irrigation should be performed with a low and constant pressure [9]. To avoid apical extrusion, the flow rate must be adjusted to the size and the design of the needle [23]. With a G30 cannula, delivering 5 mL NaOCl should last for at least 38 s (<8 mL/min).

Syringe irrigation has some limitations, and therefore, alternative irrigant delivery systems and supplementary irrigation methods are available. In vitro, GentleWave and EndoVac, two dynamic irrigation systems, produced no apical irrigant extrusion, whereas irrigant delivery with open-ended cannulas led to occasional extrusion [16]. However, further studies are needed to evaluate alternative irrigant delivery systems, such as sonic and ultrasonic activation, concerning apical irrigant extrusion in teeth with open apices.

## 5. Conclusions

Within the limitations of this in vitro study, it can be concluded that syringe irrigation using 30 G cannulas with open-ended and side-vented needle tip designs entails little risk of apical irrigant extrusion in immature teeth when a 5 mL syringe is used, and irrigant delivery takes 40 s or more. Owing to their diameter, 25 G cannulas ought to be used with caution. To reduce the likelihood of apical irrigant extrusion, delivering 5 mL of irrigant with a 5 mL syringe and a 25 G cannula should last at least 30 s. Under experimental conditions, side-vented cannulas showed a tendency to produce higher apical pressures compared with open-ended cannulas.

## Figures and Tables

**Table 1:** Results of all measurements with the size of the needle, needle tip design, TL, plunger force, duration, volume, maximum, mean, 95% confidence interval, and percentage of exceedance of the critical threshold per group (*n* = 10 per group).

Size	Type	TL (mm)	Force (N)	Duration (Seconds)	Volume (mL)	Max (mbar)	Mean (mbar)	95% CI (mbar)	Rate of Exceedance
30 G	Open-ended	0	10	30	0.79	1.48	0.30	0.21–0.38	0%
			20	10	0.73	2.50	1.01	0.68–1.34	0%
			40	10	1.55	6.64	1.49	1.10–1.87	0%
			80	5	1.60	7.97	4.38	4.06–4.70	10%
		−1	10	30	0.80	1.41	0.37	0.10–0.57	0%
			20	10	0.70	1.88	0.89	0.61–1.18	0%
			40	10	1.63	3.67	1.48	1.27–1.69	0%
			80	5	1.60	8.59	3.31	2.62–4.00	10%
		−2	10	30	0.60	1.17	0.29	0.12–0.45	0%
			20	10	0.80	1.88	0.82	0.49–1.15	0%
			40	10	1.68	3.98	2.04	1.60–2.48	0%
			80	5	1.60	7.42	2.89	2.00–3.78	0%
		−4	10	30	0.82	1.17	0.29	0.14–0.45	0%
			20	10	0.60	3.05	0.96	0.70–1.22	0%
			40	10	1.72	4.45	1.79	1.54–2.05	0%
			80	5	1.60	8.05	2.92	2.33–2−51	10%
	Side-vented	0	10	30	0.88	2.42	1.23	0.93–1.53	0%
			20	10	0.46	1.88	0.84	0.56–1.12	0%
			40	10	1.27	3.52	1.57	1.26–1.88	0%
			80	5	1.20	5.70	2.11	1.97–2.24	0%
		−1	10	30	0.74	1.64	0.59	0.32–0.87	0%
			20	10	0.48	3.91	1.05	0.78–1.32	0%
			40	10	1.28	3.52	1.46	1.17–1.74	0%
			80	5	1.40	8.13	3.70	3.31–4.09	20%
		−2	10	30	0.83	2.58	0.91	0.54–1.28	0%
			20	10	0.53	2.11	1.13	0.90–1.36	0%
			40	10	1.20	4.84	1.73	1.40–2.06	0%
			80	5	1.20	12.42	5.34	4.51–6.18	60%
		−4	10	30	0.80	2.50	0.74	0.48–0.99	0%
			20	10	0.70	2.03	1.22	0.98–1.46	0%
			40	10	1.43	5.00	2.01	1.73–2.29	0%
			80	5	1.40	10.23	4.88	4.40–5.36	60%
25 G	Open-ended	0	10	15	1.73	3.20	0.95	0.52–1.37	0%
			20	15	4.13	4.61	1.57	1.32–1.83	0%
			40	5	2.46	9.22	5.15	4.66–5.64	70%
			80	5	3.60	14.38	9.47	9.07–9.86	100%
		−1	10	15	1.75	1.72	0.39	0.20–0.59	0%
			20	15	3.96	3.98	2.07	1.72–2.42	0%
			40	5	2.32	8.91	4.50	4.27–4.72	30%
			80	5	3.60	13.91	7.71	7.22–8.20	100%
		−2	10	15	1.79	2.42	0.64	0.37–0.91	0%
			20	15	3.99	3.44	1.90	1.61–2.19	0%
			40	5	2.27	11.09	4.63	4.36–4.19	60%
			80	5	3.60	15.55	8.65	7.80–9.51	100%
		−4	10	15	1.75	1.48	0.48	0.34–0.62	0%
			20	15	4.12	4.45	2.30	1.95–2.64	0%
			40	5	2.20	23.13	4.13	3.77–4.50	60%
			80	5	3.60	40.46	19.28	16.29–22.27	100%
	Side-vented	0	10	20	4.37	2.66	1.48	1.27–1.69	0%
			20	10	4.19	8.44	4.20	3.75–4.66	30%
			40	5	3.17	23.53	9.22	8.14–10.31	100%
			80	4	3.80	28.36	18.08	17.03–19.14	100%
		−1	10	20	4.50	3.20	1.94	1.65–2.22	0%
			20	10	3.99	10.16	4.84	4.14–5.54	40%
			40	5	3.18	22.42	10.63	9.11–12.15	100%
			80	4	3.70	29.53	17.47	15.51–19.43	100%
		−2	10	20	4.66	3.98	2.12	1.78–2.46	0%
			20	10	4.03	10.31	5.49	4.61–6.38	90%
			40	5	3.34	22.81	9.03	8.39–9.68	100%
			80	4	3.70	39.22	24.29	22.16–26.42	100%
		−4	10	20	4.90	3.44	2.00	1.74–2.26	0%
			20	10	4.17	10.94	5.97	5.38–6.56	50%
			40	5	3.80	22.66	9.47	8.71–10.24	100%
			80	4	3.90	29.38	17.79	15.89–19.69	100%

**Table 2:** Recommended irrigation procedure to reduce the risk of apical irrigant extrusion in teeth with an open apex.

Size	Type	TL (mm)	Force (N)	Duration (s)
**30 G**	Open-ended	0 to −4	10–40	≥30
**30 G**	Side-vented	0 to −4	10–40	≥38
**25 G**	Open-ended	0 to −4	10–20	≥18
**25 G**	Side-vented	0 to −4	10	≥21

## Data Availability

The dataset generated and analyzed during the current study is available from the corresponding author on request.
